# Advances of the multifaceted functions of PSTPIP2 in inflammatory diseases

**DOI:** 10.3389/fimmu.2024.1492878

**Published:** 2024-11-26

**Authors:** Shaohui Geng, Bohan Hu, Yiwei Guan, Yijin Jiang, Zixuan Shu, Chen Li, Guangrui Huang

**Affiliations:** ^1^ School of Life Sciences, Beijing University of Chinese Medicine, Beijing, China; ^2^ School of Chinese Materia Medica , Beijing University of Chinese Medicine, Beijing, China; ^3^ Department of Rheumatology, Fangshan Hospital, Beijing University of Chinese Medicine, Beijing, China

**Keywords:** PSTPIP2, inflammatory diseases, osteomyelitis, inflammation, SAPHO syndrome

## Abstract

The complex interaction between the immune system and autoinflammatory disorders highlights the centrality of autoimmune mechanisms in the pathogenesis of autoinflammatory diseases. With the exploration of PSTPIP2, it has been discovered to play an inhibitory role in immune diseases, suggesting its potential utility in the research and treatment of rheumatic diseases. This review outlines the mechanisms of PSTPIP2 in chronic multifocal osteomyelitis (CMO), rheumatoid arthritis (RA), synovitis-acne-pustulosis-hyperostosis-osteitis (SAPHO) syndrome, liver diseases, renal diseases, pressure ulcer sepsis and diabetic obesity. The mechanisms include inhibiting the IL-1β inflammatory responses, NF-κB, ERK phosphorylation etc., promoting Erβ, and modulating the polarization of macrophage to prevent the inflammatory diseases. This review summarized current findings and offered perspectives on future research directions, laying a foundation for applying of PSTPIP2 in inflammatory diseases.

## 1 Introduction

As research into immune inhibitory proteins progresses, proline-serine-threonine phosphatase-interacting protein 2 (PSTPIP2), a member of the Fes/CIP4 homology-Bin/Amphiphysin/Rvs (F-BAR) domain family, has been identified as an adaptor protein residing on the cell membrane (1–3). Serving as an immune suppressor, PSTPIP2 inhibits inflammation and mitigates the damage inflicted by the immune system on the body (4–6). Its efficacy is anchored in its modulation of mutiple signaling cascades,notablyby inhibiting the functional activity of IL-1β, modulating the chemokine CXCL2 within neutrophil granules, and suppressing the production of reactive oxygen species (ROS) via the neutrophil NOX2 NADPH oxidase. Furthermore, PSTPIP2 demonstrates its role as an immune suppressor by effectively dampening inflammation and alleviating immune-induced tissue damage through these multifaceted mechanisms. It accomplishes this by inhibiting the activity of IL-1β, fine-tuning CXCL2 within neutrophil granules, and suppressing the generation of reactive oxygen species (ROS) by neutrophil NOX2 NADPH oxidase. Additionally, PSTPIP2 exerts inhibitory effects on osteoclastogenesis. By modulating these pathways, PSTPIP2 dampens immune responses, thereby mitigating the harm inflicted on the body (7).

The damage wrought by immune responses on the human body poses a formidable clinical hurdle. This condition, characterized by abnormal immune activation, often manifests as fever, joint swelling, pain, and even deformation, along with skin redness, rash, and itching. These impairments frequently pose significant therapeutic challenges (8–12). Currently, there are no specific drugs available, and most therapies aim to alleviate these symptoms by suppressing the overall immune system of the body, which can be highly detrimental to the patient (13–16). Consequently, research into these immune-mediated disorders is of paramount importance. PSTPIP2, as a relevant protein, underscores its significance value in immune disease research by suppressing immune responses and mitigating the damage caused by the immune system to the body (17–19). While researchers are actively delving into the role of PSTPIP2 in various immune disorders, the precise mechanisms underlying its function in some of these diseases, such as its inhibition of IL-1β, remain incompletely understood. A deeper exploration of these mechanisms is inperative for advancing our comprehension of PSTPIP2’s potential therapeutic applications.

**Figure 1:**
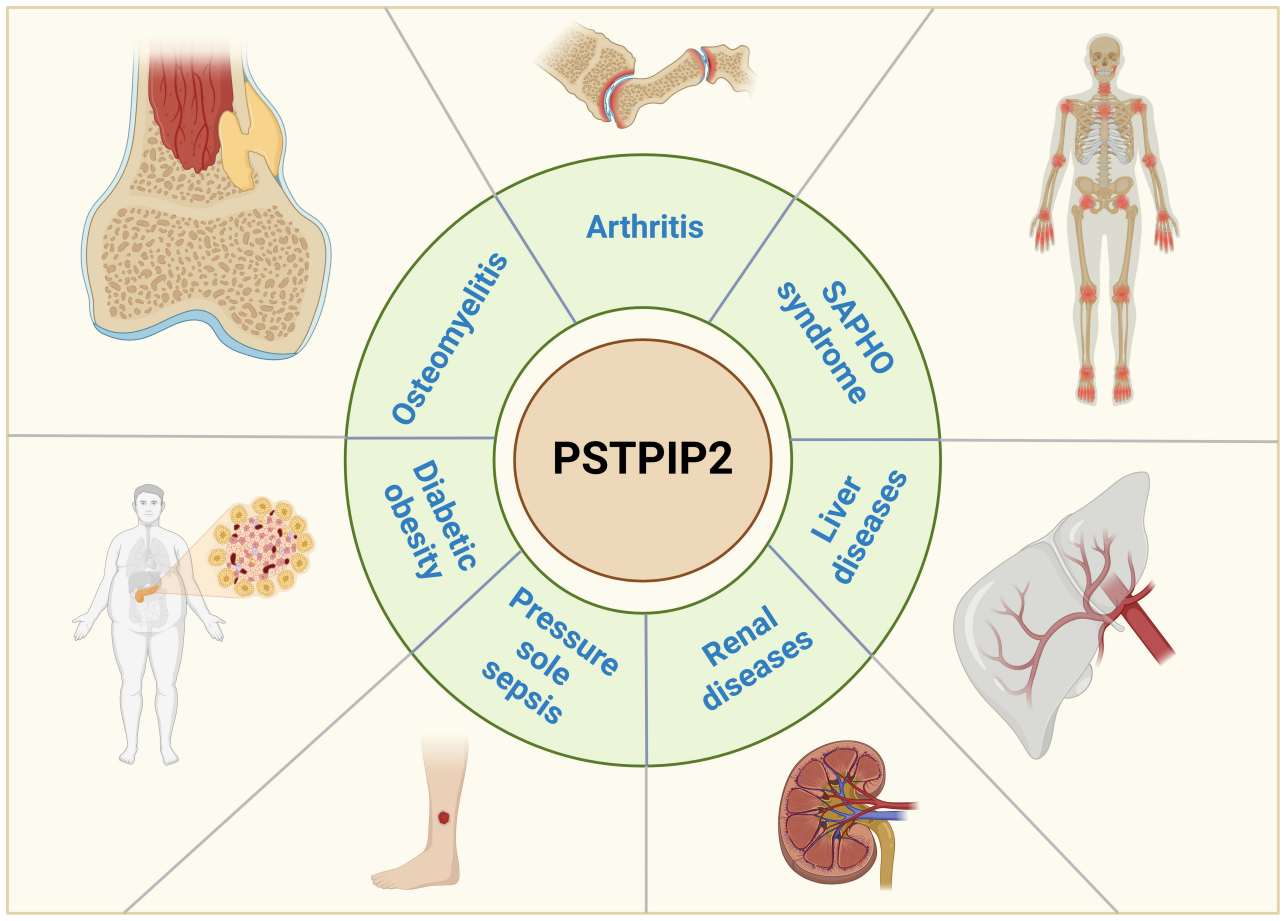
Research progress on the mechanism of action of PSTPIP2 in inflammatory diseases.

This comprehensive review offers an extensive perspective on the advancements in understanding the mechanisms of PSTPIP2 in context of autoinflammatory diseases, as vividly depicted in **Figure 1**. We systematically outline the role of PSTPIP2 in CMO, RA, SAPHO syndrome, liver diseases, renal diseases, pressure ulcer sepsis and diabetic obesity. We hope to provide insights and guidance for future research directions and drug design related to PSTPIP2 in inflammatory diseases.

## 2 Osteomyelitis

**Figure 2:**
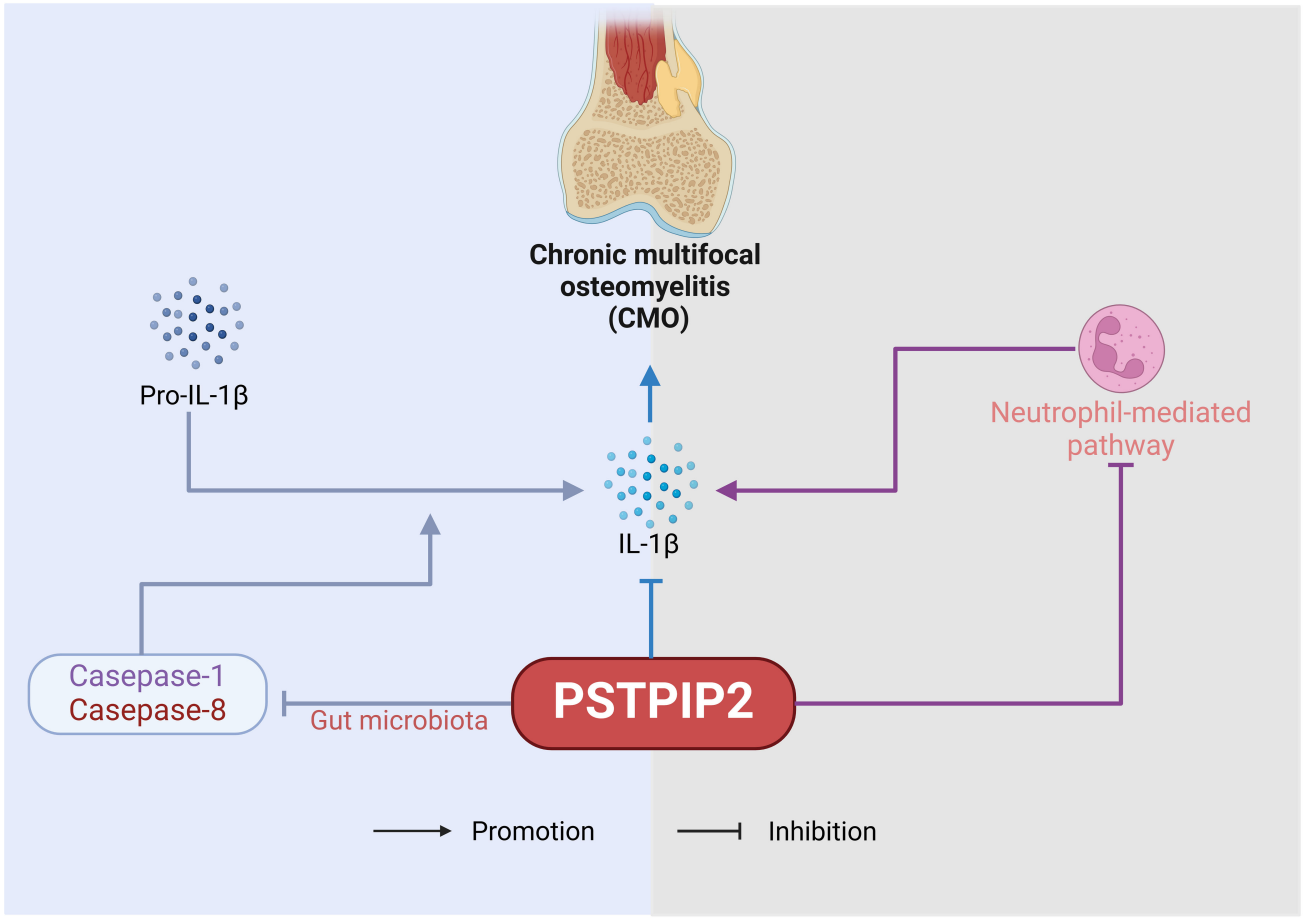
The pathogenetic role of PSTPIP2 in osteomyelitis.

Osteomyelitis, characterized by infection and bone destruction, presents with pain in the affected area, along with fever, weight loss, localized redness and swelling, and tenderness on palpation (20–23). This chapter delves into the pivotal role of PSTPIP2 protein in chronic multifocal osteomyelitis (CMO). The pathogenetic role of PSTPIP2 in osteomyelitis is shown in **Figure 2**. PSTPIP2, a crucial mediator in autoinflammatory diseases, can initiate or exacerbate the symptoms of osteomyelitis when absent or mutated. Recent research have unveiled that PSTPIP2 collaborates with the suppression of pro-inflammatory factors like IL-1β, the regulation of megakaryocyte and neutrophil functions, and the interaction with proteins such as protein tyrosine phosphatases containing a PEST domain(PEST-PTPs) to collectively inhibit inflammatory responses. Additionally, the emerging role of PSTPIP2 in modulating the gut microbiome in chronic multifocal osteomyelitis presents a novel perspective for disease treatment.

The CMO represents a distinctive subtype of osteomyelitis primarily characterized by bone pain and pyrexia. Due to the unclear pathogenesis, the treatment rate remains low (24–27). Scholars had demonstrated that the absence of the PSTPIP2 protein in animals leads to the development of autoinflammatory manifestations, suggesting a role for PSTPIP2 in controlling the onset of autoinflammatory diseases. These discoveries provide novel insights into the pathogenesis of CMO and other related autoinflammatory disorders, setting the stage for future research endeavors in this field. Ferguson, PJ et al. (28) utilized backcross strategies to localize the CMO gene and identified the presence of the PSTPIP2 gene within the region. They discovered a single base pair mutation, suggesting that mutations in PSTPIP2 may serve as a genetic determinant contributing to the autoimmune inflammatory phenotype observed in CMO mice. Similarly, Chen, TC (29)et al. induced PSTPIP2 mutations in C57BL/6 J mice through N-ethyl-N-nitrosourea mutagenesis, and these mice exhibited inflammatory responses in areas such as claws. Furthermore, Chitu, V et al. (30) found that asymptomatic PSTPIP2 (CMO) mice had an increased number of macrophage precursors in their spleens. The lack of PSTPIP2 facilitated the proliferation of macrophage progenitors and augmented the responsiveness of mature macrophages to activating stimuli. This combination effect predisposes the organism to excessive and sustained inflammatory responses, ultimately resulting in autoimmune inflammatory diseases.

### 2.1 IL-1β-mediated pathway

The inhibitory effect of PSTPIP2 on IL-1β also holds considerable significance in CMO. Drobek, A et al. (31) demonstrated that the C-terminal key tyrosine residue region of PSTPIP2 is crucial for its inhibition of IL-1β processing in neutrophils, through its binding to the inhibitory enzymes CSK and SHIP1. Furthermore, the inhibitory capacity of SHIP1 enhances this process. Similarly, Gurung, P et al. (32) revealed the combined role of IL-1β in driving the disease progression in PSTPIP2(CMO) mice. The number of IL-1 receptors (IL-1Ra) in PSTPIP2-deficient mice also impacts disease severity. Cassel SL et al. (33) demonstrated that the absence of IL-1RI in CMO mice significantly shortened the disease onset time and reduced the degree of bone lesions, indicating that controlling the number of IL-1R1 could be a potential therapeutic direction for CMO caused by PSTPIP2 deficiency. In addition, Lukens, JR et al. (34, 35) identified PSTPIP2 as a negative regulator of caspase-1-mediated autonomous IL-1β production. This finding underscores the mechanism by which PSTPIP2 exerts its anti-inflammatory effects by modulating IL-1β levels, thereby preventing the unchecked activation of inflammatory responses that could lead to autoimmune or autoinflammatory conditions.

### 2.2 Neutrophil-mediated pathway

Kralova, J et al. (10) discovered that in addition to IL-1β, PSTPIP2 negatively regulates the pathway of neutrophil NOX2 NADPH oxidase, which generates reactive oxygen species (ROS). PSTPIP2(CMO) neutrophils exhibit extremely high superoxide production in response to various stimuli, implicating dysregulated NADPH oxidase activity as a pivotal mediator of autoimmune inflammatory bone damage in PSTPIP2(CMO) mice. Furthermore, PSTPIP2 inhibits the exaggerated neutrophil responses to various stimuli induced by ROS produced by neutrophil NOX2 NADPH oxidase, thereby suppressing inflammation. Pavliuchenko, N et al. (36) used mouse strains with disrupted PEST or SHIP1 binding sites in PSTPIP2 to demonstrate that when PEST-PTPs cannot bind to PSTPIP2, it leads to dysregulation of the chemokine CXCL2 in neutrophils, causing symptomatic disease.

### 2.3 Gut microbiota-mediated pathway

Lukens, JR et al. (35) investigated the characteristics of the gut microbiota in PSTPIP2(CMO) mice and elucidated that diet-related changes in the gut microbiota composition play a pivotal role in regulating caspase-1 and caspase-8-mediated IL-1β maturation, which subsequently impacts the development of osteomyelitis in these animals. Their findings hint at the promising potential of dietary interventions aimed at modulating the gut microbiota as a therapeutic strategy for the treatment of chronic multifocal osteomyelitis (CMO) arising from PSTPIP2 deficiency. However, the specific relationship between PSTPIP2 protein and the gut microbiota remains to be further investigated.

## 3 Arthritis

**Figure 3:**
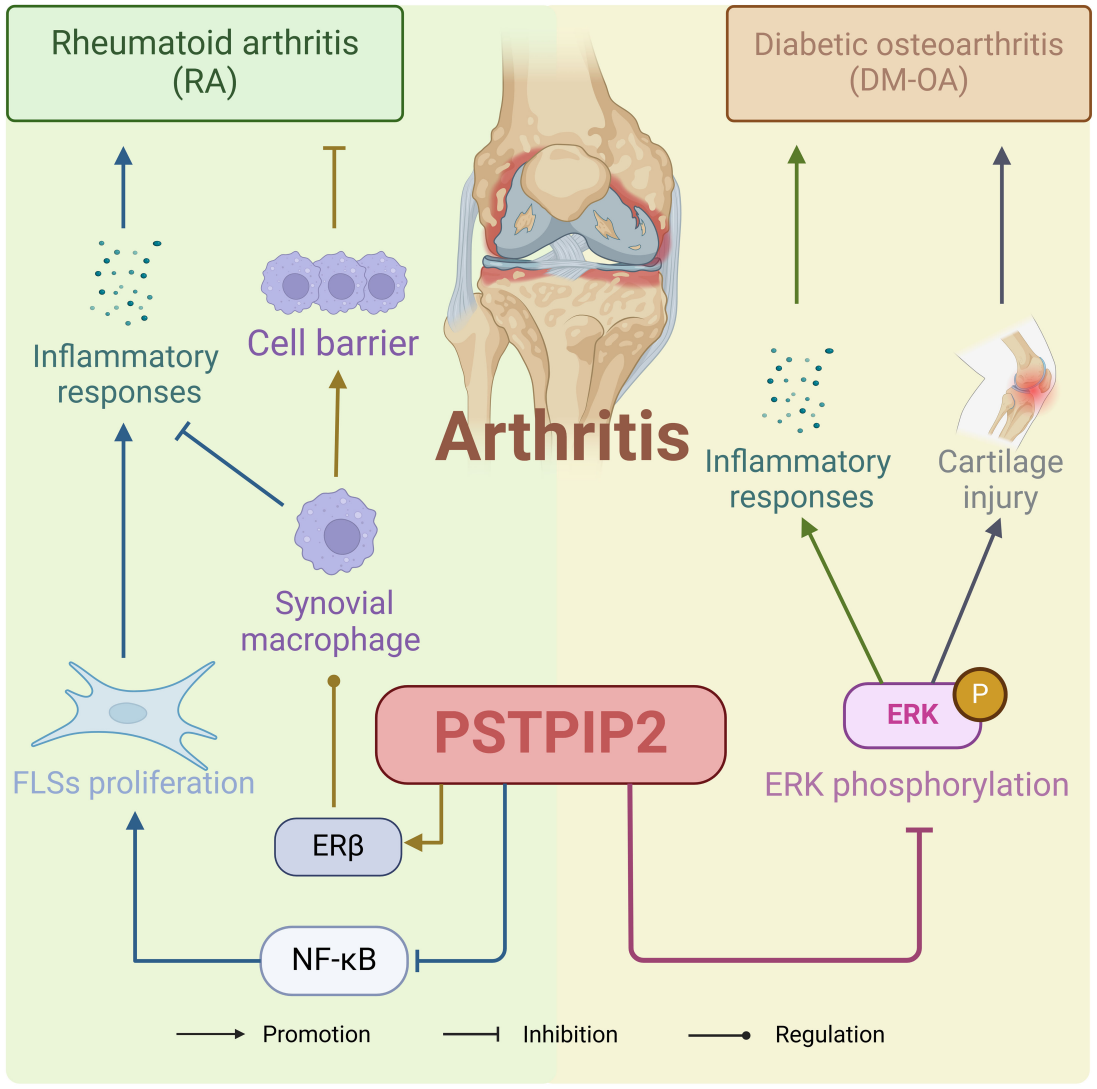
The pathogenetic role of PSTPIP2 in arthritis.

Arthritis represents a diverse group of inflammatory diseases affecting human joints and their surrounding tissues, manifesting as bone hyperplasia, and ligament tissue alterations, among other symptoms (37–40). Emerging research has shown that PSTPIP2 can inhibit osteoclast development and thereby prevent the onset of arthritis. The pathogenetic role of PSTPIP2 in arthritis is shown in **Figure 3**. Chitu, V et al. (41) delved into the mechanism underlying PSTPIP2’s regulation of osteoclast development by examining cmo models unable to express PSTPIP2 and Lupo models with PSTPIP2 dysfunction. They found that PSTPIP2 acts as a negative feedback regulator of CSF-1R signaling, inhibiting TRAP expression, and osteoclast precursor fusion, thus suppressing inflammation and osteoclastogenesis, where PSTPIP2 tyrosine phosphorylation and a functional F-BAR domain played an important role. Similarly, Tsujita, K et al. (42) discovered the complex interplay between PSTPIP2 and other F-BAR domain proteins in regulating cellular processes that impact inflammation and tissue remodeling. Sztacho, M (43) discovered that PSTPIP2 plays a role in regulating podosome assembly within the podosome/sealed dynamics monitoring mechanism.

### 3.1 Rheumatoid arthritis

Rheumatoid arthritis (RA) is a chronic autoimmune inflammatory disease (44–48), characterized by erosive arthritis with synovitis as its underlying pathological mechanism. Initial symptoms include morning stiffness, swelling, and pain in the joints, which can progressively evolve into joint deformity (49–52). Fibroblast-like synoviocytes (FLS) play a crucial role in its pathogenesis (53–56). Yao Y (3) conducted a study utilizing an arthritis animal model to investigate the function of PSTPIP2 in FLS and demonstrated that PSTPIP2 exerts inhibitory effects on FLS proliferation and inflammatory responses. Moreover, the expression mechanism of PSTPIP2 is closely related to the NF-κB signaling pathway. Grosse, J et al. (57)found that PSTPIP2 has anti-inflammatory effects in macrophages. Yao, Y (58) explored the molecular mechanism of PSTPIP2’s anti-bone erosion effects by overexpressing PSTPIP2 protein *in vivo* experiments. They discovered that PSTPIP2 regulates synovial macrophage polarization and dynamics through Estrogen Receptor Beta, forming an immune barrier (F4/80(+)PSTPIP2(hi) cell-enriched zone) at the joint, thereby controlling rheumatoid arthritis bone erosion. This suggests that locally regulating PSTPIP2 expression in the joint microenvironment may be a potential strategy for controlling rheumatoid arthritis bone erosion.

### 3.2 Diabetic osteoarthritis

Diabetic osteoarthritis (DM-OA) often affects individuals with inadequate glycemic control despite prolonged oral hypoglycemic medication and stands as a prominent cause of disability (59–62). Its primary symptoms include joint pain, stiffness, and swelling (63–65). PSTPIP2 has a mitigating effect on DM-OA and bone damage. Li, M et al. (66) investigated the potential pathways of PSTPIP2 influencing DM-OA progression by overexpressing PSTPIP2 through intra-articular injection of lentiviral vectors. They found that PSTPIP2 overexpression alleviates synovial inflammation and bone damage in DM-OA by inhibiting ERK phosphorylation. In addition, Liu, L et al. (67)also discovered PSTPIP2 overexpression caused enhanced activation of Src family kinases and subsequently reduced ERK phosphorylation, and verified that PSTPIP2 upregulation repressed megakaryocyte development in primary mouse bone marrow cells.

## 4 SAPHO syndrome

SAPHO syndrome is an uncommon condition primarily affecting bones and skin, with its primary diagnostic feature being chronic multifocal osteitis (68–71). The protein PSTPIP2 plays crucial roles in macrophage activation, neutrophil migration, and osteoclast differentiation (72). Liao HJ et al. (73) generated PSTPIP2 knockout (Pstpip2(-/-)) mice and observed that all Pstpip2(-/-) mice developed an inflammatory disease resembling SAPHO syndrome. Notably, inflamed tissues exhibited significant elevations in chemokines attracting neutrophils and IL-1β, hinting at a potential role for PSTPIP2 in innate immunity and autoinflammatory bone diseases, possibly linked to the pathogenesis of human SAPHO syndrome. Marzano, AV et al. (74) summarized that in SAPHO syndrome, the activation of PSTPIP2 inflammasomes is thought to contribute to the induction of innate immune system dysfunction. However, there are also different points of view, Hurtado-Nedelec M (75) analyzed the PSTPIP2 gene in patients with SAPHO syndrome. Compared to controls, no specific or more frequent rare variations in this gene were observed in SAPHO patients, indicating no correlation between PSTPIP2 variations and SAPHO syndrome. Therefore, further research is imperative to unravel and elucidate the underlying mechanistic connections between PSTPIP2 and SAPHO syndrome.

## 5 Liver system diseases

**Figure 4:**
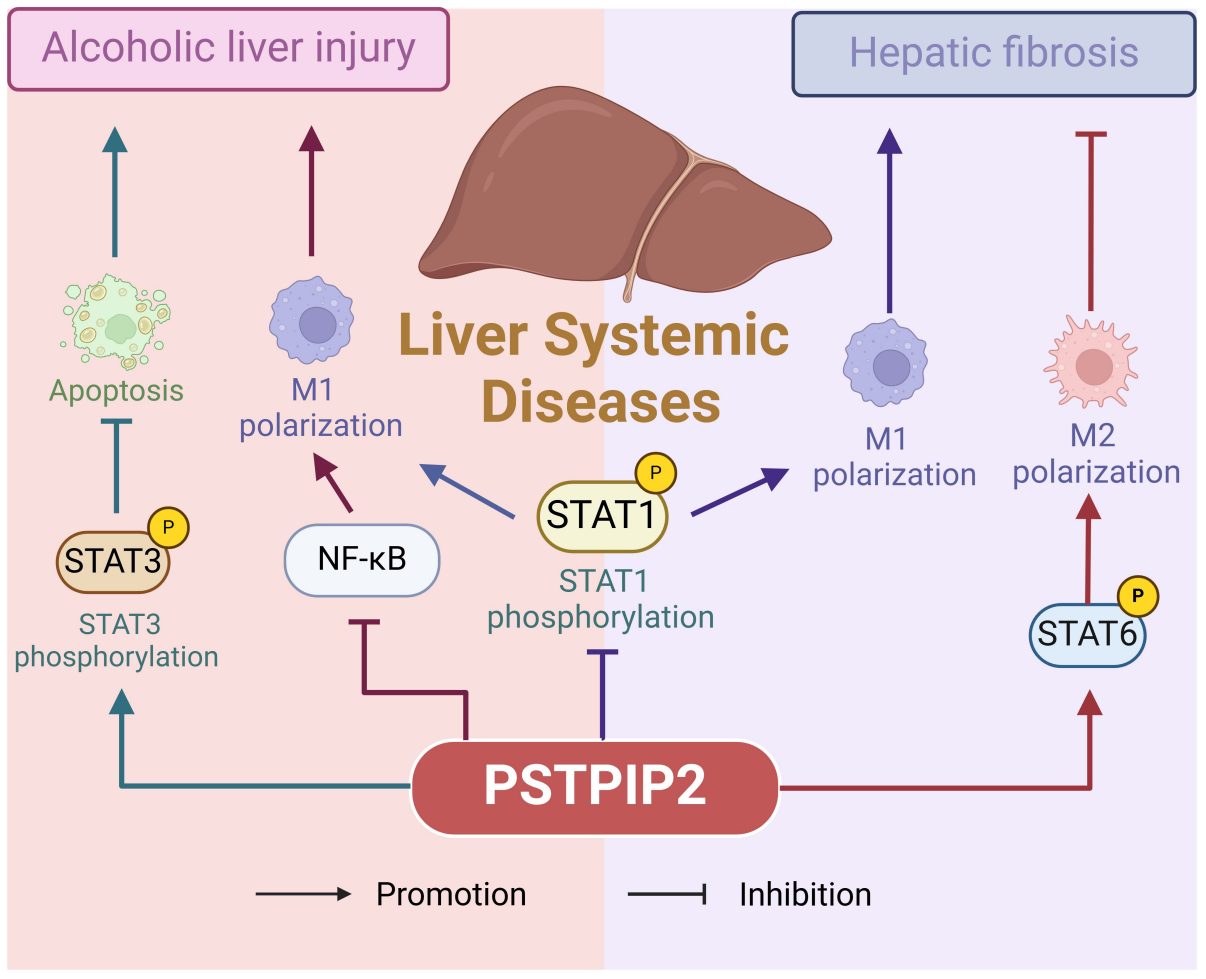
The pathogenetic role of PSTPIP2 in liver system diseases.

Liver system diseases, encompassing a range of disorders that impair liver function, such as liver injury and hepatitis, represent a complex spectrum of conditions. Liver injury is usually caused by external forces or viral infections (76, 77), whereas alcoholic liver injury (ALI) is caused by chronic excessive alcohol consumption and manifests as fatigue, anorexia, bloating, and diarrhea (78). **Figure 4** illustrates the pathogenic role of PSTPIP2 in liver system diseases. This chapter emphasizes the multifaceted nature of PSTPIP2 in liver disease, suggesting its potential application in therapeutic strategies and the need for further research.

### 5.1 Alcoholic liver injury

Liver injury, a relatively common condition, affects the liver and is characterized by varying degrees of hepatocyte damage induced by external forces or viral infections (79–82). Alcoholic liver injury (ALI), specifically, arises from prolonged and excessive alcohol consumption, manifesting with symptoms such as fatigue, anorexia, abdominal distension, and diarrhea (83–86). Yin, NN et al. (87) utilized an ethanol (EtOH)-fed mouse model and an EtOH-induced AML-12 cell model to demonstrate that PSTPIP2 regulates hepatocyte apoptosis in ALI through the signal transducer and activator of transcription 3 (STAT3) pathway. Xu, JJ et al. (88) found that in ALI, ethanol induces aberrant methylation of PSTPIP2 and elevates the expression of proteins such as DNMT3a. Furthermore, the silencing of DNMT3a significantly restored ethanol-induced low PSTPIP2 expression and inhibited ethanol-induced inflammation.

### 5.2 Liver fibrosis and hepatitis

Hepatitis is a diverse range of conditions caused by bacteria, viruses, parasites, alcohol, drugs, chemicals, and autoimmune factors, leading to impaired liver function and abnormal liver function indicators (79, 89–91). PSTPIP2 has been implicated in alleviating hepatic fibrosis and inflammation. For instance, Yang, Y et al. (92) investigated the function of PSTPIP2 in hepatic fibrosis through adeno-associated virus (AAV9)-mediated PSTPIP2 overexpression, investigating the molecular mechanisms underlying PSTPIP2-regulated hepatic fibrosis. They discovered that increased PSTPIP2 expression alleviates hepatic fibrosis and inflammation in mice by modulating macrophage polarization. However, contrasting research suggests that PSTPIP2 may promote the progression of hepatitis C. Chao, TC et al. (93) employed a lentiviral-based RNA interference (RNAi) screening approach to identify PSTPIP2 as a potential cellular factor involved in HCV replication. They further demonstrated the importance of PSTPIP2’s membrane-deforming ability in HCV replication, proposing that PSTPIP2 facilitates membrane alterations and participates in the formation of membrane webs, which are crucial for HCV replication complexes. Therefore, a more detailed investigation into the mechanisms of PSTPIP2 in different types of hepatitis is warranted.

## 6 Renal system diseases

**Figure 5:**
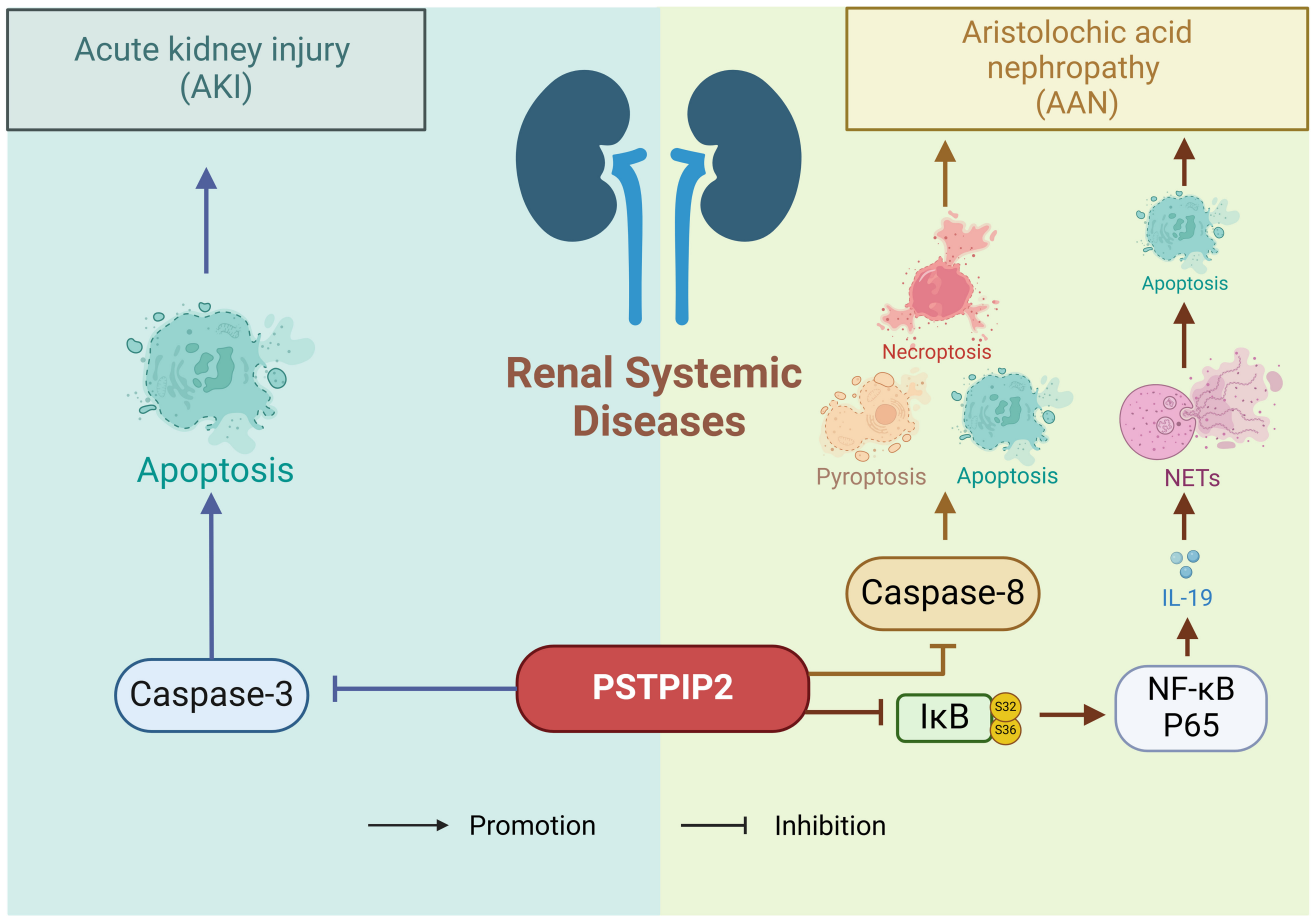
The pathogenetic role of PSTPIP2 in renal system diseases.

The kidney is a highly susceptible genitourinary organ prone to damage from trauma, spontaneous rupture, iatrogenic injuries, and other factors, resulting in symptoms such as hematuria, pain, and shock (94–96). The previous research has shown a close association between PSTPIP2 and inflammatory diseases, with histone deacetylases potentially mediating the expression of PSTPIP2. The pathogenetic role of PSTPIP2 in renal systemic diseases is shown in **Figure 5**. In the context of renal injury, Zhu, H et al. (97) conducted a study to determine the specific role of PSTPIP2 in cisplatin-induced acute kidney injury (AKI), discovering that cisplatin might silence PSTPIP2 through histone acetylation. Xu, CT et al. (98) experimentally found that histone deacetylase (HDAC)-mediated PSTPIP2 silencing may contribute to the development of aristolochic acid nephropathy (AAN). Du, CL et al. (99) demonstrated that neutrophils and neutrophil extracellular traps (NETs) play crucial roles in AAN, and proposed that therapeutic targets targeting PSTPIP2/nuclear factor (NF)-κB/IL-19/IL-20Rβ could offer novel strategies for reducing aristolochic acid I-mediated acute kidney injury and apoptosis.

## 7 Other conditions

### 7.1 Pressure sore sepsis

Pressure sore sepsis is a chronic condition characterized by deep skin breakdown accompanied by pus and bleeding (100–102). PSTPIP2 exhibits anti-inflammatory effects in pressure-sore sepsis. Wang, XX et al. (103) compared the expression levels of PSTPIP2 in peripheral blood samples from 20 patients suffering from sepsis secondary to pressure ulcers and 10 healthy controls. They found that patients with sepsis due to pressure ulcers had lower levels of PSTPIP2 in their peripheral blood. Further, lipopolysaccharide (LPS)-induced THP-1 cells expressed lesser amounts of PSTPIP2 compared to untreated control cells. Additionally, the transfection of PSTPIP2 resulted in reduced levels of IL-6, IL-1β, and TNF-α, while also inhibiting the activation of the NF-κB signaling pathway. These findings collectively suggest that PSTPIP2 is associated with the severity of pressure sore sepsis and exerts anti-inflammatory effects, implying potential anti-inflammatory roles of PSTPIP2 in other skin-related inflammatory conditions that remain to be explored.

### 7.2 Diabetic obesity

Diabetic obesity refers to a condition where individuals have both diabetes and obesity, often leading to increased health risks and complications such as cardiovascular diseases, hypertension, and metabolic disorders (104–106). In the realm of diabetic obesity, PSTPIP2 also alleviates obesity-related tissue inflammation in diabetic mice. Xu, J et al. (107) established a diabetic mouse model through a high-fat diet (HFD) and discovered that PSTPIP2 promotes M2 macrophage polarization via activation of PPARγ, thereby mitigating obesity-related adipose tissue inflammation and insulin resistance in diabetic mice. These findings hint at the potential of PSTPIP2 as a therapeutic target for diabetes, presenting a novel therapeutic trajectory for managing this condition.

## 8 Outlook

This review comprehensively examines the mechanisms of PSTPIP2 in diverse diseases, with a particular emphasis on its advancements in osteomyelitis, arthritis, SAPHO syndrome, hepatic diseases, renal diseases, and other inflammatory conditions. As a pivotal immunomodulatory protein, PSTPIP2 exhibits broad-spectrum immunosuppressive effects through multiple pathways, including inhibition of IL-1β, modulation of neutrophil and macrophage activity, and regulation of gut microbiota.

While considerable progress has been made in elucidating the mechanisms of PSTPIP2 in various diseases, numerous challenges persist and avenues for future research remain unexplored. Firstly, the intricate mechanisms of PSTPIP2 in diverse diseases remain incompletely understood, particularly its dual and sometimes paradoxical roles in different liver disease manifestations. For instance, while PSTPIP2 exhibits protective effects against acute liver injury (ALI) and fibrosis, it paradoxically facilitates viral replication in hepatitis C. A profound understanding of these mechanisms is paramount for the development of targeted therapeutic strategies that can harness the immunomodulatory potential of PSTPIP2 without exacerbating undesirable effects. Secondly, the intricate relationship between PSTPIP2 and the gut microbiota merits thorough investigation. The gut microbiota serves as a crucial modulator of immune responses, and its intricate interplay with PSTPIP2 may offer novel insights into potential dietary interventions for disorders related to PSTPIP2 deficiency. By exploring this interaction, there is potential to uncover dietary interventions or probiotics that could be employed to address PSTPIP2 deficiency-related disorders, thereby providing patients with an alternative or adjunctive therapeutic approach. Moreover, the development of PSTPIP2-targeted therapeutics remains a crucial research direction. Currently, there are no commercially available drugs specifically targeting PSTPIP2, despite its promising therapeutic potential across a range of immune-mediated diseases. Progress in this area will necessitate overcoming substantial scientific and technical challenges, including the identification of specific binding sites on PSTPIP2 and the development of molecules capable of effectively modulating its activity without eliciting adverse effects. Additionally, future studies should explore the potential synergies between PSTPIP2-targeted therapies and current treatment modalities for immune-mediated diseases. Specifically, the integration of PSTPIP2 modulators with conventional immunosuppressants could potentially augment treatment efficacy and mitigate the risk of adverse events.

In conclusion, by conducting in-depth investigations into the mechanisms underlying PSTPIP2 and addressing the associated challenges, we can pave the way for innovative therapeutic strategies for a diverse array of immune-mediated diseases. These endeavors will not only elevate treatment efficacy and enhance patients’ quality of life but also contribute significantly to a deeper understanding of the intricate interplay between the immune system and various diseases. This holistic approach is imperative for advancing the field of immunomodulatory therapies and improving clinical outcomes for patients with immune-mediated disorders.

## Glossary

SAPHO: synovitis-acne-pustulosis-hyperostosis-osteitis
RA: rheumatoid arthritis
ROS: reactive oxygen species
PSTPIP2: proline-serine-threonine phosphatase-interacting protein 2
F-BAR: Fes/CIP4 homology-Bin/Amphiphysin/Rvs
CAO: chronic aseptic osteomyelitis
ENU: N-ethyl-N-nitrosourea
SNP: single nucleotide polymorphism
CRMO: chronic recurrent multifocal osteomyelitis
MCP-1: monocyte chemoattractant protein-1
CSK: C-terminal Src kinase
ERK: extracellular signal-regulated kinase
PEST-PTPs: protein tyrosine phosphatases containing a PEST domain
SHIP1: Src homology 2 domain-containing inositol-5'-phosphatase 1
CSK: C-terminal Src kinase
CMO: chronic multifocal osteomyelitis
FLS: fibroblast-like synoviocytes
DM-OA: Diabetic osteoarthritis
ALI: Alcoholic liver injury
STAT3: signal transducer and activator of transcription 3
AAV9: adeno-associated virus
RNAi: RNA interference
AKI: acute kidney injury
HDAC: histone deacetylase
AAN: aristolochic acid nephropathy
NETs: neutrophil extracellular traps
LPS: lipopolysaccharide
HFD: high-fat diet
IL-1Ra: IL-1 receptors
